# Parasite Zoonoses and Wildlife: Emerging Issues

**DOI:** 10.3390/ijerph6020678

**Published:** 2009-02-13

**Authors:** R.C. Andrew Thompson, Susan J. Kutz, Andrew Smith

**Affiliations:** 1 World Health Organization Collaborating Centre for the Molecular Epidemiology of Parasitic Infections, School of Veterinary and Biomedical Sciences, Murdoch University, Murdoch, WA 6150, Australia; E-mail: Andrew.Smith@murdoch.edu.au; 2 Faculty of Veterinary Medicine, University of Calgary, 3330 Hospital Drive NW, Calgary, Alberta, Canada, T2N 4N1; E-mail: skutz@ucalgary.ca

**Keywords:** Wildlife, zoonoses, parasitic infections, emergence, biosecurity, *Sarcoptes*, *Giardia*, *Echinococcus*, *Toxoplasma*, *Leishmania*, *Trypanosoma*

## Abstract

The role of wildlife as important sources, reservoirs and amplifiers of emerging human and domestic livestock pathogens, in addition to well recognized zoonoses of public health significance, has gained considerable attention in recent years. However, there has been little attention given to the transmission and impacts of pathogens of human origin, particularly protozoan, helminth and arthropod parasites, on wildlife. Substantial advances in molecular technologies are greatly improving our ability to follow parasite flow among host species and populations and revealing valuable insights about the interactions between cycles of transmission. Here we present several case studies of parasite emergence, or risk of emergence, in wildlife, as a result of contact with humans or anthropogenic activities. For some of these parasites, there is growing evidence of the serious consequences of infection on wildlife survival, whereas for others, there is a paucity of information about their impact.

## 1. Pathogens in Wildlife

Wildlife have long been recognized as potential sources for emerging infectious diseases in humans and domestic animals, and wildlife diseases have historically gained attention primarily when they were considered a threat to agricultural systems and the economic, social, or physical health of humans [1–3]. In fact, the potential impacts of infectious diseases on wildlife populations have often been overshadowed by the apparently more pressing anthropocentric issues. However, today, there is a rapidly evolving understanding of the ecology of infectious diseases in wildlife, including a new appreciation of the impact that infectious diseases can have on the dynamics and sustainability of wildlife populations. In particular, the serious threat that disease can impose on genetically impoverished endangered wildlife species is increasingly recognized, as is the importance of preserving biodiversity in wildlife ecosystems to prevent and control the emergence or re-emergence of diseases [1–6].

**Figure 1.**
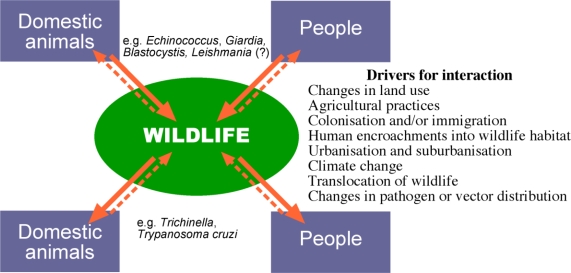
Pathogen flow among wild and domestic host-pathogen systems. Solid arrows depict spill-over from the natural host to a novel or accidental host, dashed arrows indicate spill-back into the original host population. The relative frequency of the spill-over and spill-back events can vary, but both are expected to increase as a result of human activity [Figure drawn by Russ Hobbs].

As we look forward, increased interactions between humans and their domestic animals and wildlife are anticipated and will facilitate the ongoing spill-over situations from domesticated reservoir populations to sympatric wildlife. In addition, just as spill over from domesticated animals, and humans, might represent a serious threat to wildlife, these same wildlife populations might then act as reservoirs and/or amplifiers of emerging and exotic diseases for domestic animals and humans [7]. Much of what we understand about the spill-over of pathogens from domestic systems to wildlife has been concerned with microbial and viral pathogens. By contrast, the often less dramatic, but perhaps equally important eukaryote parasites (i.e. protozoa, helminths and arthropods) have received little attention. Furthermore, with the exception of non-human primates, most attention has focussed on the spill-over of pathogens from domestic animals to wildlife and little consideration has been given to pathogens of humans spilling over into wildlife. Here, we focus on parasites of ‘domestic’ origin and their potential impact on wildlife. In particular, we examine the emerging scenario of humans as a source of new parasite infections in wildlife and, as a consequence, the establishment of ‘spill-back’ reservoirs of these zoonotic parasites in wildlife (Figure 1).

## 2. Spill-Over *vs* Spill-Back

Understanding the direction of flow in parasite life cycles is important in determining how wildlife reservoirs of parasitic diseases are established.

The transmission and establishment of zoonotic parasites of wildlife in domestic animals and humans is well recognized with parasitic diseases such as trichinellosis (*Trichinella*) and Chagas disease (*Trypanosoma cruzi*). In these cases, wildlife are naturally infected, usually with little impact on their health, but they serve as important reservoirs of infection for cycles in domestic animals [8] that may spill-over as a consequence of human encroachment on wildlife habitats, hunting or changes to agricultural practices. The converse, where the principal cycles for maintaining a parasite are domestic but which can spill-over to wildlife, are less well defined, particularly for parasites (Figure 1). As we detail below, the creation of such wildlife reservoirs indirectly as a result of human behaviour or directly from human hosts, can lead to a potential for spill-back to humans and domestic animals.

## 3. Transmission of Parasites from Humans to Wildlife

Recent research has demonstrated that wildlife reservoirs can be established through parasite infections transmitted directly from human hosts.

The concept that parasites for which humans are a natural reservoir can spill-over directly to wildlife is not widely recognized; neither is the fact that when this happens, new reservoirs of potential public health significance may be established in wildlife.

### 3.1. Sarcoptes/Sarcoptic Mange

Sarcoptic mange, or scabies, is a well-recognized threat to the health and sometimes existence of endangered or isolated wildlife populations [9]. In Australia, sarcoptic mange caused by *S. scabei* var. *wombati* occurs throughout the range of the common wombat in southeast Australia and has the potential both to dramatically reduce the local abundance of wombats and threaten the survival of small isolated populations [10]. There is strong, albeit controversial evidence that humans and domestic dogs were the recent source of the variant of *Sarcoptes scabei* that has severely affected wombat populations in Australia [11]. *Sarcoptes scabei* has also been reported as a cause of clinical disease in human-habituated gorillas, and it is thought that transmission may occur among gorillas, people, and livestock [9].

### 3.2. Giardia/Beaver Fever

**Figure 2.**
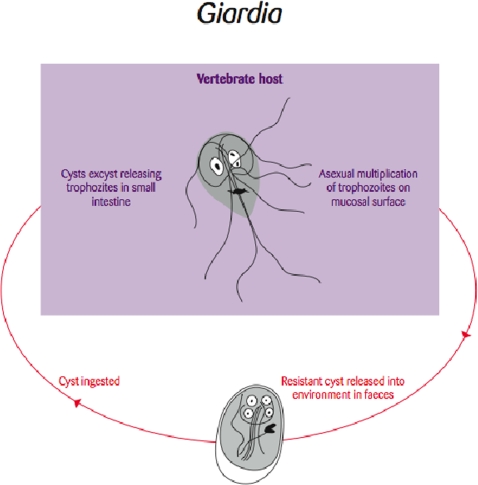
Life cycle of the flagellate protozoan parasite *Giardia* [Re-drawn by Gareth Parsons from an original figure by Russ Hobbs].

Molecular typing of the common enteric protozoan *Giardia* (Figure 2) has drastically altered our thinking of this parasite as a wildlife pathogen that is spilling over into humans. Most evidence to date suggests that more often than not the parasite spills over from domestic cycles into wildlife populations. Further, once infected, these wildlife populations may maintain the parasites and serve as an ongoing spill-back reservoir for humans.

It is often a common ‘knee-jerk’ reaction when parasites with zoonotic potential are found in wildlife that they represent a threat to public health [12] as a reservoir and potential source of infection for humans [13]. Indeed, this was the case when WHO initially listed the common enteric protozoan parasite *Giardia* as a zoonosis over 25 years ago as a result of epidemiological observations suggesting that giardiasis in campers in Canada was caused by drinking stream water contaminated with *Giardia* from beavers [13]. No one thought to ask the question of where the beavers got their *Giardia* infections from until only beavers downstream from a sewage works were found to be infected. With the subsequent application of molecular tools, it has been confirmed that beavers are susceptible to zoonotic strains of *Giardia* [13]. The question now is: are they victim or villain with respect to human giardiasis?

A similar situation has been reported in non-human primates for which there is a growing literature of the invasion of human pathogens into wild populations [14–16]. For example, it was suggested that the finding of *Giardia* and the co-habiting enteric protozoan *Cryptosporidium* in mountain gorillas in the Bwindi Impenetrable National Park Uganda was thought to indicate enhanced contact with humans and/or domestic livestock. This was confirmed when rangers and their cattle were found to be infected with *Giardia* and that the genotype was the same as that recovered from the gorillas [15].

Muskoxen (*Ovibos moschatus*) are indigenous to the arctic tundra of Canada and Greenland and have been translocated to areas in Alaska, USA, Russia, Norway and Sweden. These animals are well adapted to their northern environment, and tend to have a relatively simple parasite fauna. Recent surveys on the biodiversity and impacts of parasites in Arctic ungulates described *Giardia duodenalis*, Assemblage A, the zoonotic genotype, in muskoxen [16]. This unexpected finding (a novel strain, or the livestock strain, was predicted) raises many interesting questions regarding the origin and epidemiology of this parasite in humans and wildlife in this Arctic ecosystem. In particular, is this a pathogen initially introduced to muskoxen by humans? Is *Giardia* now maintained as a sylvatic cycle in muskoxen (or other wildlife species on the island) independent of humans? Does the *Giardia* from muskoxen spill-back into humans?

The permanent human population of Banks Island is restricted to one small community of ~ 120 humans, many of whom spend extended periods of time ‘on-the-land’ hunting, fishing and drinking directly from the water bodies. Additionally, < 100 tourists visit the island annually for outdoor recreational opportunities. Muskoxen and humans tend to concentrate around the lush river valleys, an ideal setting for interspecies sharing of a faecal-orally water-borne parasite. Other sources of ongoing dispersal of the parasite include the disposal of offal from commercial muskox harvests on the land (in the past) and, more recently, on the sea ice, raising questions about the strain and source of the *Giardia* detected in seals in this region which are known to be susceptible to zoonotic strains of *Giardia* of human origin [16].

Another little explored area with respect to *Giardia* in wildlife are the impacts at individual and population levels. In experimentally and naturally infected sheep, *Giardia* reduced rates of weight gain, impaired feed efficiency and decreased carcass weight [17,18]. In cattle, *Giardia* is commonly found alone or in combination with other pathogens as a cause of calf diarrhoea, which can have economic significance [19]. The impact of *Giardia* on the health and production (body condition, fecundity and pelage) of free-ranging ungulates, including muskoxen, remains unknown.

Similarly, in Australia, marsupials are commonly infected with *Giardia* but until recently, it was not known to what species or strain(s) of *Giardia* they were susceptible. Studies on the Quenda (*Isoodon obesulus*), a common widespread species of bandicoot in southern Australia, demonstrated that they were infected with a novel, genetically distinct form of *Giardia*, so different to what has been described from humans and other animals, that it probably represents a distinct species [20]. The *Giardia* isolates genotyped from Quenda in their natural habitats have all proved to be the novel strain. However, when Quenda were trapped and examined on a farm, they were found to be infected with ‘domestic’ strains of *Giardia* normally found in livestock and humans. Presumably this reflects the susceptibility of Quenda to other strains of *Giardia*, as with the case of beavers in North America. This case study raises questions regarding the pathogenicity of non-host adapted strains of *Giardia* in naïve wildlife hosts. Additionally, it also raises the question of competition between co-habiting ‘strains’ of *Giardia* [21] and whether in this case, and perhaps in other species of wildlife, zoonotic strains of *Giardia* can out-compete the host-specific wildlife strains.

## 4. Transmission of Parasites between Wildlife and Domestic Animals

In some cases wildlife reservoirs are established through parasite infections transmitted from domestic animals but as a result of human activity.

### 4.1. Echinococcus: Hydatid Disease

Emerging issues with the pathogenic tapeworm (cestode) parasite *Echinococcus* illustrate very well how anthropocentric issues overshadow the potential impacts that infectious diseases may have on wildlife populations. For example, when the distribution of the species *E. multilocularis* in the USA increased as a consequence of the translocation of foxes for hunting, the public health threat was considered to be the most important issue [22,23]. Ironically, in Switzerland, the anti-rabies vaccination program in foxes, with a clear benefit to humans, resulted in a 4-fold increase in fox numbers from 1980 through 1995 - this has resulted in an emerging epidemic of alveolar hydatid disease in humans [24].

**Figure 3.**
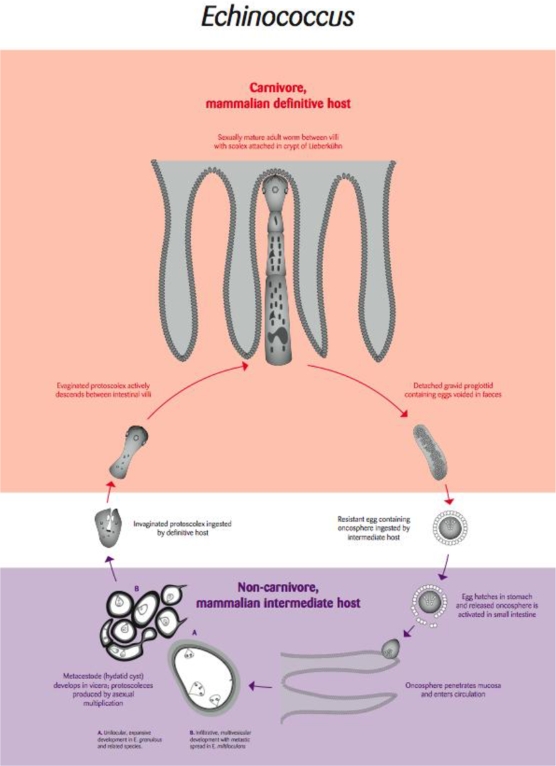
Life cycle of the tapeworm parasite *Echinococcus* [Re-drawn by Gareth Parsons from an original figure by Russ Hobbs].

Hydatid disease is a systemic cystic infection caused by the larval stage of parasites of the genus *Echinococcus*. The parasite is maintained in a two-host life cycle involving carnivorous definitive hosts in which the adult, sexually reproducing cestode develops in the small intestine (Figure 3). Intermediate hosts, which may include humans, acquire infection by accidentally ingesting embryonated (infective) eggs that have been released into the environment in the faeces of infected definitive hosts (Figure 3). This results in the development of the larval cystic stage, usually in the lungs or liver, of the intermediate host. In the case of *E. multilocularis* the larval stage can behave like a metastatic invasive tumour, whereas with other species of *Echinococcus*, the larval cystic stages are not invasive and form space occupying fluid-filled cysts.

*E. multilocularis* is principally maintained in a wildlife cycle involving foxes and arvicolid rodents. The emerging issues associated with man-made increases in fox populations in the USA and Europe are exacerbated by anthropogenic landscape changes such as deforestation and agricultural practices which have led to more favourable conditions for the intermediate hosts especially arvicolid rodents [25]. Little attention has been given to the impact of the fast growing invasive larval stage of *E. multilocularis* in naïve rodent hosts which may suffer increased mortalities as a direct result of infection or enhanced susceptibility to predation [26]. *E. multilocularis* relies on its definitive host consuming infected intermediate hosts and increased susceptibility to predation is expected to enhance parasite persistence [26].

The life cycle of another species of *Echinococcus*, *E. canadensis* involves wolves and large cervids in North America and Scandinavia [27]. In susceptible cervids such as moose in which the hydatid cysts preferentially develop in the lungs, there is evidence that infection predisposes infected moose to predation by wolves, and that this might be an important factor in the persistence of wolf populations [28–31]. Thus it appears that an ecological balance has been struck where this species of *Echinococcus* is maintained in a natural sylvatic cervid-carnivore cycle. However, in Western Canada, human activity has resulted in the spill-over of this sylvatic cycle of *E. canadensis* into farmed elk that is believed to involve domestic dogs as the result of inadequate disposal of the offal from the elk [29].

**Figure 4.**
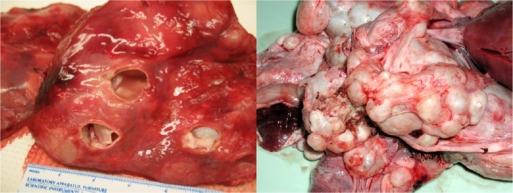
Hydatid cysts in the lungs of a moose (left) and kangaroo (right; photo courtesy of Russ Hobbs).

However, *Echinococcus* in cervids is distinct from the more widely distributed ‘domestic’ species of *Echinococcus*, *E.granulosus*, which infects livestock and dogs, and to which humans are susceptible to infection with the cystic stage [27]. *E. granulosus* was introduced into Australia with sheep during early settlement in the late 1700’s and now appears was the source of widespread infections with the larval cystic stage in many species of macropod marsupials (wallabies and kangaroos) throughout the Australian mainland. In marsupials, as with *E. canadensis* in moose, *E. granulosus* has a predilection for the lungs and can result in massive infections (Figure 4).

Dingoes hunt a range of macropodids from small wallabies to large kangaroos and it has long been considered that hydatid cysts in the lungs of wallabies could weaken the animal and thus render them more easily captured by dingoes [31–37]. Recent studies suggest that hydatid disease reduces effective lung volume in wallabies by ∼ 55% in males and ∼ 70–80% in females [34]. These authors consider that such reductions impact the fitness of the animals to a degree seldom seen in sheep where infection is widely recognized as asymptomatic [34]. Barnes and colleagues [34] also consider that, apart from enhancing susceptibility to predation, the presence of hydatid disease might be fatal and a threat to the survival of endangered small macropod species that exist in small isolated colonies with small home ranges.

Thus, from an ecological perspective, the recent introduction of *Echinococcus* into Australia with domestic livestock has resulted in the establishment of a sylvatic (wild) life cycle that can affect predator-prey relationships, as well as host survival directly [37]. Such impacts could both be significant in Australia, where many species of marsupial are under threat. In addition, the establishment of a dingo-macropod cycle, which effectively maintains parasite transmission, also acts as a ‘spill-back’ reservoir of infection for sheep and cattle and is a major problem for control strategies that focus on education and husbandry activities to break the domestic ‘dog-sheep’ cycle [37].

### 4.2. Toxoplasma/Toxoplasmosis

**Figure 5.**
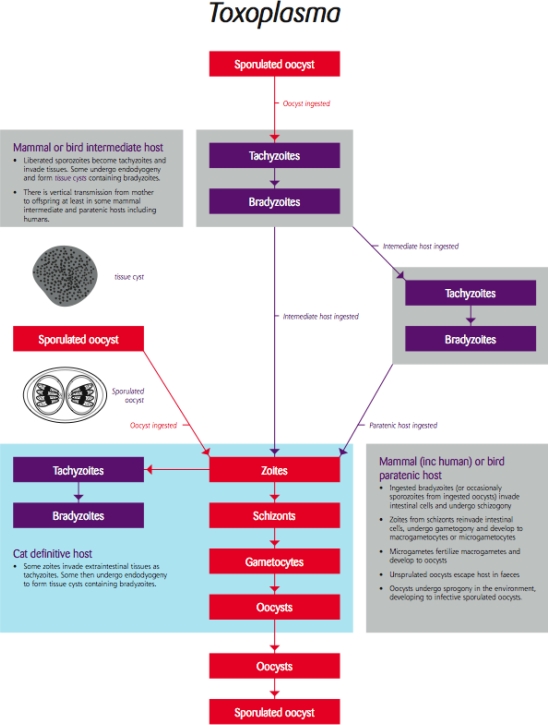
Life cycle of the coccidian protozoan parasite *Toxoplasma* [Re-drawn by Gareth Parsons from an original figure by Russ Hobbs].

Despite toxoplasmosis being one of the most common parasitic infections in the world, it is a rare disease [38]. Most species of mammals and birds are susceptible to infection with this protozoan parasite and can act as intermediate hosts. Infection is usually systemic resulting in a short period of rapid multiplication in various tissues followed by the establishment of tissue cysts in the muscles and brain (Figure 5). Tissue cysts form in response to the host’s immune response and are effectively a dormant phase in the parasite’s life cycle in terms of causing overt, symptomatic disease, causing no harm unless re-activated as a consequence of a lowered immune response, and can persist for the life of the host [39].

Parasite stages in the tissues are transmitted only if ingested (predation or scavenging) or if passed vertically from mother to foetus (Figure 5). Felids (usually cats) are the definitive host and the parasite undergoes sexual multiplication and development in the intestine releasing environmentally resistant infection of the developing foetus may result in abortion or damage to the newborn but this does not always occur and there is a growing body of opinion that vertical transmission may be a mechanism for maintaining *Toxoplasma* in animal populations [40].

Wildlife are susceptible to infection with *Toxoplasma* which may lead to chronic asymptomatic infection, severe clinical consequences and death, or subtle effects on the nervous system such as risky behaviour that can increase susceptibility to predation [41,42]. However, although there is a widespread distribution of *Toxoplasma* infection in wildlife there are relatively few reports of overt clinical disease in nature with most reports relating to animals in captivity. This serves to emphasise that certain factors, such as stress induced by concurrent infection, nutritional factors, or captivity, can compromise the immune system and predispose subclinically infected wildlife to clinical toxoplasmosis.

Human encroachment into wildlife habitats can also have a role in the spread of *Toxoplasma* to wildlife. For example, in the USA, outbreaks of toxoplasmosis in sea otters are thought to be due to terrestrial water run-off contaminated with domestic cat faeces [43,44]. In Australia, humans and their domestic cats are thought to have introduced the protozoan parasite *Toxoplasma* where it is now widespread, affecting numerous species of native wildlife, particulalry marsupials. There is also anecdotal evidence that it could be associated with die-offs in some marsupial populations [45,46]. This could be as a result of infected animals dying as a result of acute infection but perhaps more likely is that the behavioural changes associated with chronic, latent infection reduce anxiety thus enhancing the success of predation [47].

On the face of it, these seem to be reasonable examples of ‘domestic’ parasites affecting wildlife, although the stories are far from complete. Recent application of molecular, genotyping tools has demonstrated that although a domestic feline origin may account for some *Toxoplasma* infections in sea otters, most isolates of *Toxoplasma* recovered from sea otters are a novel strain or genotype not yet found in domestic cats [43,44]. Similarly, *Toxoplasma* from Australian wildlife has not yet been genotyped and novel strains might be found in Australian native fauna. These examples serve to reinforce the value of molecular tools for the genetic characterization of parasites from tissue or environmental samples, and how they will increasingly have a major impact on our understanding of the interaction between domestic and wildlife cycles of many parasites.

## 5. The Potential for Disease Emergence in Wildlife

### 5.1. Leishmania and Trypanosoma

**Figure 6.**
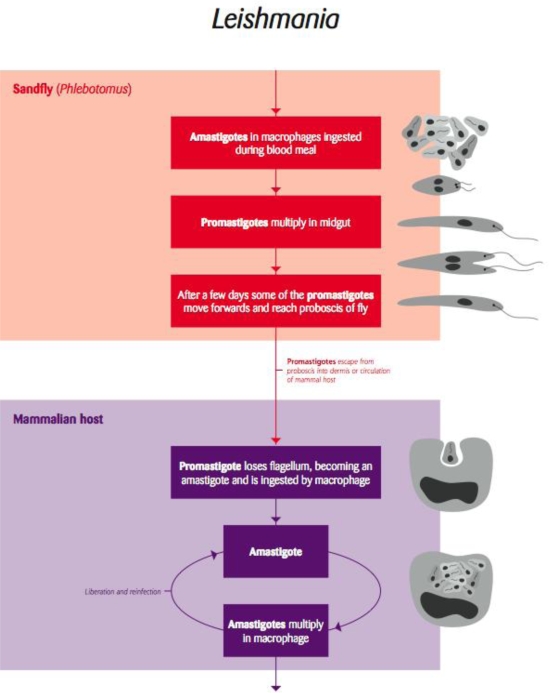
Life cycle of the arthropod borne flagellae protozoan parasite *Leishmania* [Redrawn by Gareth Parsons from an original figure by Russ Hobbs].

*Leishmania* is a vector-borne trypanosomatid protozoan parasite transmitted by sandflies (Figure 6). This intracellular pathogen of mammals consists of numerous species and subspecific variants that affect a variety of wildlife mammalian hosts. In addition, humans and domestic dogs are susceptible to infection with several species, which often results in serious disease.

*Leishmania* has a broad geographical distribution but SE Asia and Australasia have never been considered as endemic areas. Therefore, the recent discovery of *Leishmania* in kangaroos in the Northern Territory of Australia [48] raises a number of issues. Initially, and perhaps not surprisingly, media and government focused in different ways on speculation that the kangaroos could be a source of infection to humans [48].

However, a systematic investigation of the parasites isolated from the lesions of affected kangaroos, including molecular characterisation of the isolated parasites, demonstrated that they did belong to the genus *Leishmania* but not to any species so far described [48]. This indicates that kangaroos and possibly other native mammalian fauna in Australia harbour a novel species of *Leishmania* that has perhaps evolved over thousands of years and adapted to its marsupial host. Although the pathogenic significance of this species to wildlife is not known and may be minimal to animals in the wild, it raises the question of how *Leishmania* is transmitted between kangaroos. Presumably, there are species of sandflies capable of acting as vectors of *Leishmania* in Australia (Figure 6). If so, these sandflies could also transmit other species of *Leishmania*. Pathogenic species of *Leishmania* regularly enter Australia in infected humans or dogs from endemic areas of the world [49–51]. Until recently, it has been assumed that such infections represent a minimal biosecurity risk since Australia does not have vectors capable of transmitting the parasite. The discovery of the parasite in kangaroos demonstrates that this is not the case, and thus imported cases of *Leishmania* pose a risk of being transmitted to humans, their pets and to wildlife. Wildlife could become a significant reservoir, as well as suffer the potentially more serious clinical consequences associated with exposure to a novel introduced pathogen likely to be of human origin in view of the increasing number of introduced cases in immigrants to Australia [51].

The situation with *Leishmania* in Australian wildlife, demonstrates how little we know about systemic and blood parasites of native wildlife in this country. For example, we are only just beginning to understand the diversity of a closely related group of vector-borne trypanosomes, *Trypanosoma*, in Australian marsupials [52] their potential impact on the health of wildlife and the relationship indigenous trypanosomes may have to exotic, human pathogenic trypanosomes, that could establish a reservoir in native wildlife. Although the trypanosomes that cause disease in humans are primarily the result of spill back from wildlife reservoirs (e.g. *T. cruzi* and *T. bruci*), human activities have almost certainly been responsible for introducing trypanosomes from one wildlife population to another. Furthermore, the establishment of an exotic trypanosome cycle within Australian wildlife would be greatly facilitated if specific arthropod vectors were inadvertently introduced at the same time. For example, the introduction of flea-infested ship rats onto Christmas Island (approximately AD 1900) resulted in the spread of the pathogenic trypanosome *Trypanosoma lewisi* into the native rat population which were described as ‘morbid’ on subsequent visits and extinct within 25 years [53]. Recent DNA-based findings indicate that the native rat population was devoid of any trypanosome-like infection prior to arrival, suggesting *T. lewisi* was probably maintained initially in a reservoir of ship rats and flea vectors and subsequently spread by contact between infected fleas and naïve native rats. The spread of *T. lewisi* may have been enhanced if ectoparasites associated with native rats were biologically capable of acting as more than just mechanical vectors, but considering the gregariousness of ship rat fleas in general, their involvement was probably not essential.

## 6. Concluding Remarks and Perspectives for the Future

Here we have highlighted an important yet neglected and emerging issue that has direct relevance both to public health and to the conservation of wildlife worldwide. The impact of spill-over of human parasites to naïve species of wildlife is not well understood yet such spill-overs are likely to increase in the future, establishing novel spill-back reservoirs of potential public health and economic significance, as well as threatening wildlife. Research to date has only scratched the surface. Our current understanding of many parasitic zoonoses is inadequate, in part because it lacks reliable information on parasite identification which is essential for making epidemiological determinations [40]. The examples we have highlighted would not have been identified without the application of molecular tools that enabled species and subspecific characterisation of the parasites concerned. Future research that uses such molecular tools will greatly enhance our understanding of the ecology of parasitic diseases including parasite flow among domestic, wild, and human hosts. It will greatly enhance our understanding of host specificity, particularly with respect to the potential host range of novel/introduced pathogens, and provide information on life history characteristics, such as environmental persistence and vectorial capacity. Our knowledge of the evolutionary biology of parasites will also benefit and as such allow predictions to be made on virulence characteristics and the likely impact of control strategies.
